# Utilizing Evolved Extramedullary Techniques for Conventional Knee Arthroplasty in Post-traumatic Knee Arthritis With Retained Hardware: A Retrospective Analysis

**DOI:** 10.7759/cureus.79402

**Published:** 2025-02-21

**Authors:** Chih-Wei Chang, Yu-Kai Tseng, Yen-Nien Chen, Chyun-Yu Yang

**Affiliations:** 1 Orthopedics, College of Medicine, National Cheng Kung University, Tainan, TWN; 2 Joint Reconstruction Center, Orthopedics, National Cheng Kung University Hospital, Tainan, TWN; 3 Orthopedic Surgery, Kuo General Hospital, Tainan, TWN; 4 Orthopedic Surgery, Pingtung Veterans General Hospital, Pingtung, TWN; 5 Institute of Biomedical Science, National Sun-Yat Sen University, Kaohsiung, TWN; 6 Physical Therapy, Asia University, Taichung, TWN; 7 Orthopedics, Cheng Kung University Hospital, Tainan, TWN

**Keywords:** conversion total knee arthroplasty, extramedullary, knee arthroplasty, post-traumatic knee arthritis, retained hardware, techniques

## Abstract

Introduction

For post-traumatic knee arthritis with retained implants, performing conventional primary total knee arthroplasty (TKA) can be challenging due to altered bony structures or mechanical obstructions. Without current computer-aided navigation, we can still perform conventional TKA by extramedullary means according to the parallelism and the reference to inherent anatomical landmarks. In this study, we aim to present a novel extramedullary method applied during conventional total knee arthroplasty (TKA) and the clinical outcomes observed in our series.

Methods

Between April 2014 and March 2021, patients with post-traumatic knee arthritis and retained hardware scheduled for TKAs at our institute were included. All the index procedures were performed with primary cemented TKA prostheses by the same surgeon. After the proximal tibial cut perpendicular to the tibial shaft, the following distal femur cut was executed according to the parallelism between these cuts. Certain interfering hardware located on the metaphyseal area, like screws or staples, were removed as needed. In addition, most of the conventional principles and techniques in traditional knee replacement were followed. Clinical outcomes, including perioperative blood loss, transfusion rate, inpatient stay, knee functional score, and radiographic evaluation, were recorded and reviewed.

Results

Within a period of eight years, there were 12 patients with a mean age of 67.8 years old enrolled. Overall, there was a good restoration of optimal mechanical alignment after surgery, and the mean hip-knee-ankle (HKA) angle was 180.2^o^ compared to their preoperative deformity (HKA 174.0^o^ ± 10.4^o^). Clinical assessments according to the Knee Society score showed good knee scores (87.1), functional scores (87.1), and motion arc (106.7^o^). No patient required allogenic blood transfusion or prolonged stays (range: five to eight days). In the last outpatient visit, no radiographic implant failure was found, and no surgical site-specific complications were reported after clinical follow-ups for at least two years.

Conclusions

Our results suggest that post-traumatic knee arthritis with remnant hardware can be safely and effectively treated with conversion TKAs according to the suggested extramedullary (EM) techniques in this study. With minimal removal of interfering hardware, reduced surgical trauma as well as operation time, helps to achieve short-term clinical benefits, including reduced blood loss, inpatient stay, faster recovery, and even restoration of optimal limb alignment.

## Introduction

Total knee arthroplasty (TKA) is an effective surgical intervention for advanced post-traumatic knee arthritis when conservative treatments fail [1-3]. However, complications such as infection, stiffness, and compromised wound healing have been documented in the literature [3,4]. Furthermore, it is a challenging and technique-demanding task to perform TKAs on post-traumatic knees with retained implants [1]. Considering the technical difficulty, retained hardware, especially the femoral nails or plates, frequently obstructs the guiding rods in conventional intramedullary-guided TKA. This may necessitate the staged removal of remnant implants prior to the index procedure [5]. Even though excessive surgical procedures prior to knee replacement can increase the likelihood of subsequent arthrofibrosis and infection [4,6]. By contrast, simultaneous arthroplasty and removal of hardware is an alternative, but it may increase the risks of skin necrosis, prolonged operation time, infection, and even periprosthetic fractures by the stress risers from the extraction of prior implants [4,7].

In the given scenario, advanced technologies such as computer-assisted navigation (CAN) and premade patient-specific blocks (PSB) have been proposed as beneficial [8,9]. However, these advantages come with additional costs for the required equipment and devices [5,9,10]. After reviewing the literature on TKAs with retained hardware, we recognize the importance of executing the bony cuts using reliable extramedullary (EM) referencing methods [5,8,11]. To obtain acceptable alignments as well as surgical accuracy, we proposed practical and reproducible principles for EM referencing for critical bone cuts in the proximal tibia and distal femur. The aim of this study was to evaluate the clinical effectiveness of our EM referencing method for conversion TKAs in post-traumatic knees with retained hardware.

The initial draft of this study was previously posted on a preprint server, Research Square, on February 27, 2020. Since then, it has been revised to include updated concepts and additional cases.

## Materials and methods

This retrospective study was at the National Cheng Kung University Hospital, Tainan, Taiwan. Between April 2014 and March 2021, patients suffering from arthritic knees and retained periarticular hardware underwent their conversion TKAs by the same surgeon using similar cemented prostheses that were identified. These cases were reviewed after Institutional Review Board approval was obtained.

**Figure 1:**
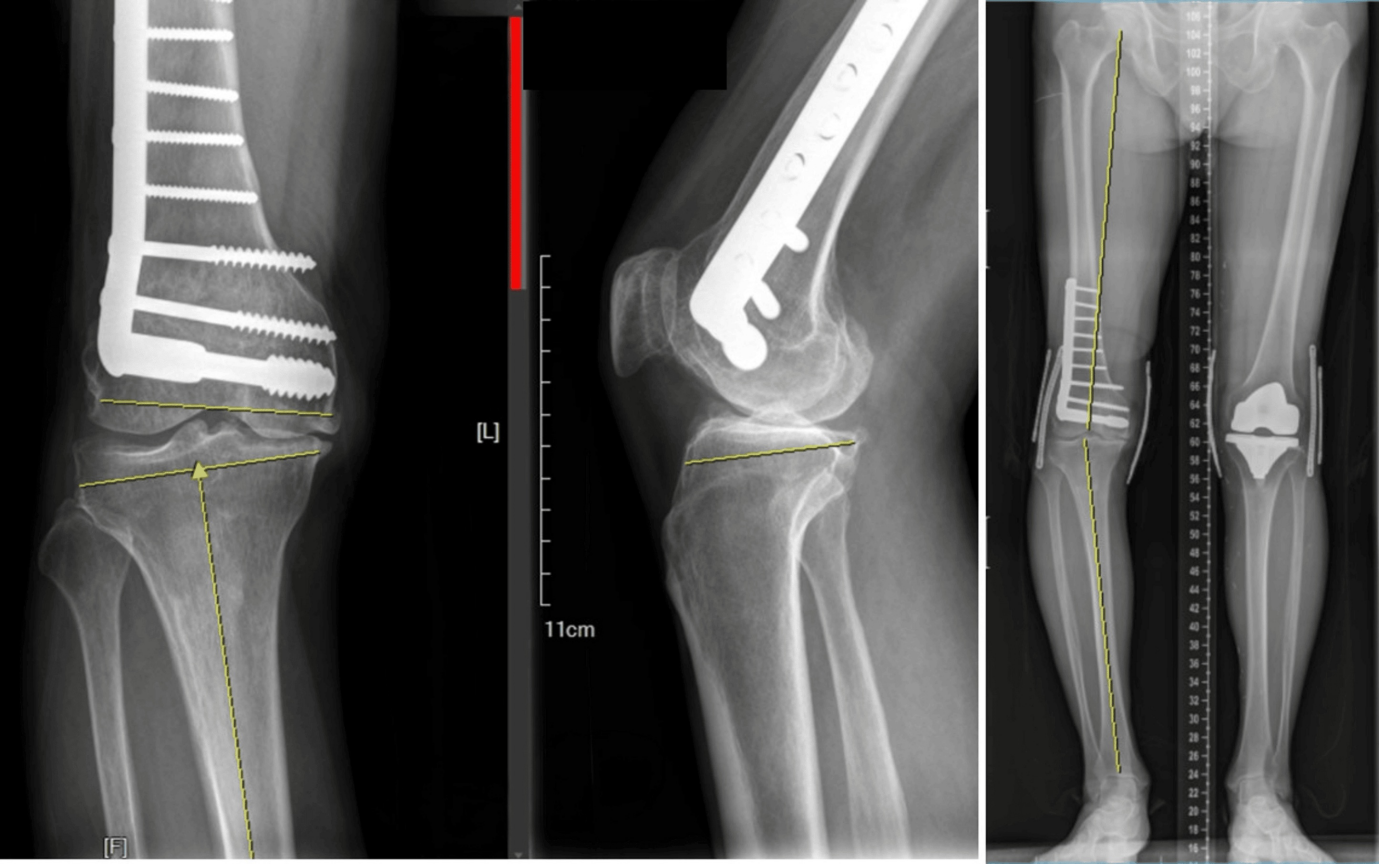
Preoperative radiographic planning Critical bone cuts on the proximal tibia and distal femur are planned using images, including standing AP, lateral projections, and long-leg films (scanogram). The necessity of removing prior hardware is also evaluated. For planning the proximal tibial cut, a horizontal line perpendicular to the tibial shaft is drawn according to the depth set within 2 mm of the most damaged part on the tibial plateau (AP view), as is our practice in conventional primary TKA. The determined shape helps the surgeon control the orientation of their oscillating saw, usually thin in the medial part and thick in the lateral part in cases of genu varum. Additionally, the lateral projection helps the surgeon assess the posterior tibia slope and adjust the inclination of the saw accordingly during the osteotomy. Typically, the osteotomized fragment is thick anteriorly and thin at the back when there is a positive tibial slope. Extreme left image: anterior-posterior (AP) view; image in the center: lateral view; extreme right image: scanogram TKA: total knee arthroplasty

Like other primary TKAs in our daily practice, standard radiographs, including the anterior-posterior, lateral views of the knee, and the long-standing scanogram, were obtained before surgery. In addition to assessing the preoperative mechanical axis and angular deformity, the potential hardware hindering the bony preparation or implantation was identified within the region between the proximal tibia and distal femur cuts drawn on the images (Figure 1).

Preoperative knee function as well as range of motion were evaluated and recorded. All patients had their knee function evaluated using the Knee Society Score (KSS) [12], and their range of motion was measured during preoperative and postoperative outpatient follow-ups.

Due to the obstructed or deformed medullary canal by the prior orthopedic implants, these surgeries employed the same EM referencing method instead of the conventional intramedullary cutting jig. Under general anesthesia, all the operated knees were approached via the same medial skin incision and parapatellar arthrotomy in knee flexion. A pneumatic tourniquet, ranging from 280 to 320 mmHg, was used to achieve a bloodless field during surgery. After joint exposure with adequate soft tissue release as well as removal of marginal osteophytes, partial removal of the obstructing screws or hardware was performed.

**Figure 2:**
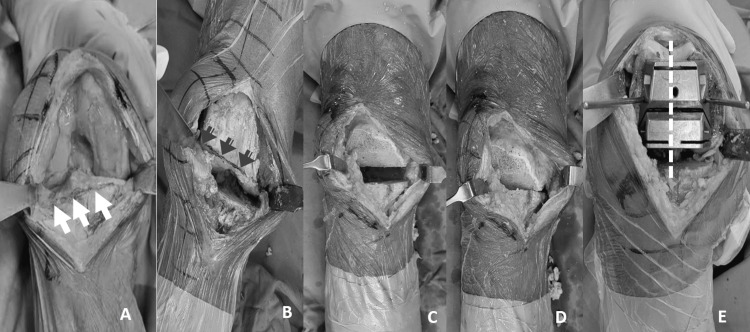
Tibia-cut-based extramedullary referencing technique A: horizontal cut line on the proximal tibia, referenced to the tibial shaft and drawn with electrocautery (indicated by white arrows); B: parallel cut line on the distal femur, drawn with electrocautery and referenced to the previous proximal tibia cut (indicated by gray arrows) while pulling the shin part with even distraction; C: parallelism between the proximal tibial and distal femur cuts, verified under knee distraction; D: coronal alignment of the lower limb, verified by complete bony contact between the proximal tibial and distal femur cuts; E: rotation of the femoral component, simply determined by the commercial four-in-one cutting jig, referencing the Whiteside line (white dotted line).

For the critical proximal tibial cut, a horizontal line perpendicular to the tibial shaft, set within 2 mm from the most damaged part, was marked on the tibial plateau using electrocautery at 90 degrees of knee flexion (Figure 2A). The osteotomy was then made along this horizontal cut line. The following distal femoral cut was executed based on the principle of parallelism. A parallel line referencing the mentioned proximal tibial cut was marked on the distal femur under even distraction without skewed limb alignment (Figure 2B). The distal femur cut was then made with a steady oscillating saw. To avoid neurovascular injury, this cut might be performed in steps. It was started along the drawn line in knee extension to avoid the skew. Once the saw blade had proceeded through the anterior half of the distal femur condyles, the remaining cut was completed under knee flexion to protect the neurovascular structures behind. After these two critical cuts, the overall coronal alignment of the lower extremity was thus determined. The required parallelism and even bony cuts were verified again with the knee extended and in distraction or bony contact (Figures 2C, 2D). The remaining femoral cuts, including the anterior, posterior, and chamfering cuts, were executed using the commercial “four-in-one” cutting jigs referencing the antero-posterior axis (Whiteside’s line) (Figure 2E) [13]. 

All the kneecaps were resurfaced. After evaluating the implant orientation, restored mechanical axis, soft tissue balancing, and deforming stresses, similar cemented prostheses with posterior-stabilized design (U2 knee®: United Orthopedic Co., Taiwan; Genesis II®: Smith & Nephew, New Zealand; Scorpio NRG®: Stryker, NJ, USA) were implanted in all knees. After surgery, all patients were managed according to an established clinical pathway for primary TKA in our ward, including the rehabilitation protocol, pain control strategy, and fluid supplements. Prophylaxis for thromboembolic events was conducted using 500 mg of intravenous lysine acetylsalicylic acid per day for the first three postoperative days, along with active ankle pumping and early ambulation. Early knee motion and ambulation were encouraged in all patients, even on the same day of the operation. In addition to a stable wound, active knee flexion of at least 90 degrees and stable independent ambulation were the criteria for discharge.

Clinical assessment

Radiographic data, hospitalization records, and all postoperative information were retrieved from inpatient electronic healthcare records. Radiological assessments followed a standardized protocol, with standing radiographs taken when the knee was in maximum extension, ensuring the patella was facing forward and both hips and ankles were visible on the film (Figure 1). Lateral radiographs were captured at 30° of knee flexion. These standard radiographs facilitated the determination of hip-knee-ankle (HKA) angles. Hospitalization data encompassed tourniquet time, mean hemoglobin drop, and length of stay. Postoperative data included emergency department (ED) visits, readmissions, site-specific complications, and mortality.

Due to the small patient sample size, the statistical analysis lacked sufficient power to detect differences in outcomes or complications.

## Results

Within eight years, 12 severe arthritic knees with retained hardware, including two males and 10 females, were treated with conversion TKA using one-stage surgical intervention. Demographic features and clinical results of these cases are presented in Tables 1, 2. The mean age of these subjects was 67.8 years (ranging from 57 to 77 years). A varus deformity was present in the majority of our arthritic knees before surgery, except for three valgus knees. The mean pre-operative HKA angles were 174.0^o^ (ranging from 157^o^ to 191^o^). Among the 12 knees, a variety of periarticular hardware was included, ranging from staples used in ligament reconstruction to nails or plates used in fracture fixation. All knees were implanted with cemented prostheses typically used in primary TKAs, and none of them required special augments like wedges or stems.

**Table 1:** Patient demographic data BMI: body mass index; LCP: locking plate; DCS: dynamic condylar screw; DCP: dynamic compression plate; HKA: hip-knee-ankle angle; TKA: total knee arthroplasty; var: varus; val: valgus

Case No.	Age	Gender	BMI (kg/m^2^)	Side	Retained hardware	Injury to TKA (years)	Preop HKA (deg)
1	67	F	28.0	R	Tibial nail	2	10.0/var
2	72	F	23.5	R	Femoral nail	14	23.0/ var
3	60	F	34.0	R	Tibial LCP	8.5	4.5/ var
4	68	M	23.3	R	Femoral DCS	3	8.6/var
5	69	F	37.8	L	Femoral/tibial LCP	6	11.4/val
6	57	F	21.4	R	Femoral staples	5	10.5/var
7	76	M	24.4	R	Femoral & tibial staples	40	9.9/var
8	66	F	31.3	L	Femoral & tibial screws	15	12.1/var
9	68	F	35.2	R	Femoral LCP	4	7.5/var
10	73	F	24.7	R	Femoral/tibial LCP	3	5.8/val
11	61	F	26.2	L	Tibial DCP	4	10.3/val
12	77	F	33.2	L	Femoral LCP	4	14.0/var

**Table 2:** Perioperative data *maximum Hb drop; HKA: hip-knee-ankle U2 knee®: United Orthopedic Co., Taiwan; Genesis II®: Smith & Nephew, New Zealand; Scorpio NRG®: Stryker; NJ, USA

Case No.	Prostheses implanted/design/ PE thickness (mm)	Ischemic time (min)	Removed hardware	Hb drop* (g/dL)	Inpatient stay (day)	Postop HKA	Follow-up duration (mon)	Transfusion
1	United, U2; PS; 9 mm	73	Screws x 4	2.6	6	1.0/var	69	Nil
2	United, U2; PS; 9 mm	86	Screws x 7	3.2	7	0.4/var	45	Nil
3	Stryker, NRG; PS; 9 mm	100	Screws x 7	3.6	8	2.1/val	41	Nil
4	United, U2, PS; 9 mm	80	Nil	3.6	6	5.0/val	34	Nil
5	Stryker, NRG; PS; 9 mm	131	Screws x 6	1.6	6	1.6/val	76	Nil
6	United, U2; PS; 9 mm	160	Nil	3.2	6	2.2/var	65	Nil
7	S & N, Genesis II; PS; 9 mm	117	Staple x 1	4.9	6	3.0/val	36	Nil
8	United, U2; PS; 9 mm	98	Screw x 1	2.6	6	0.5/val	31	Nil
9	United, U2; PS; 9 mm	94	Screw x 1	1.6	5	1.3/var	24	Nil
10	United, U2; PS; 11 mm	76	Screws x 5	2.2	5	0.3/val	31	Nil
11	United, U2; PS; 9 mm	95	Screws x 3	1.9	9	1.0/val	18	Nil
12	S & N, Genesis II; PS; 9 mm	104	Screws x 4	2.2	6	6.0/var	43	Nil

The mean ischemic time for the operation was 101.2 minutes (ranging from 73 to 160 minutes). During hospitalization, the mean maximum hemoglobin drop was 2.77 g/dL (ranging from 1.6 to 4.9 g/dL), and no patients required allogenic blood transfusions under adequate fluid supplements. The mean duration of their inpatient stay was 6.33 days (ranging from five to nine days). Only one patient (Case 11) with end-stage renal disease extended her hospital stay for regular hemodialysis rather than adhering to the routine discharge criteria for TKA.

**Figure 3:**
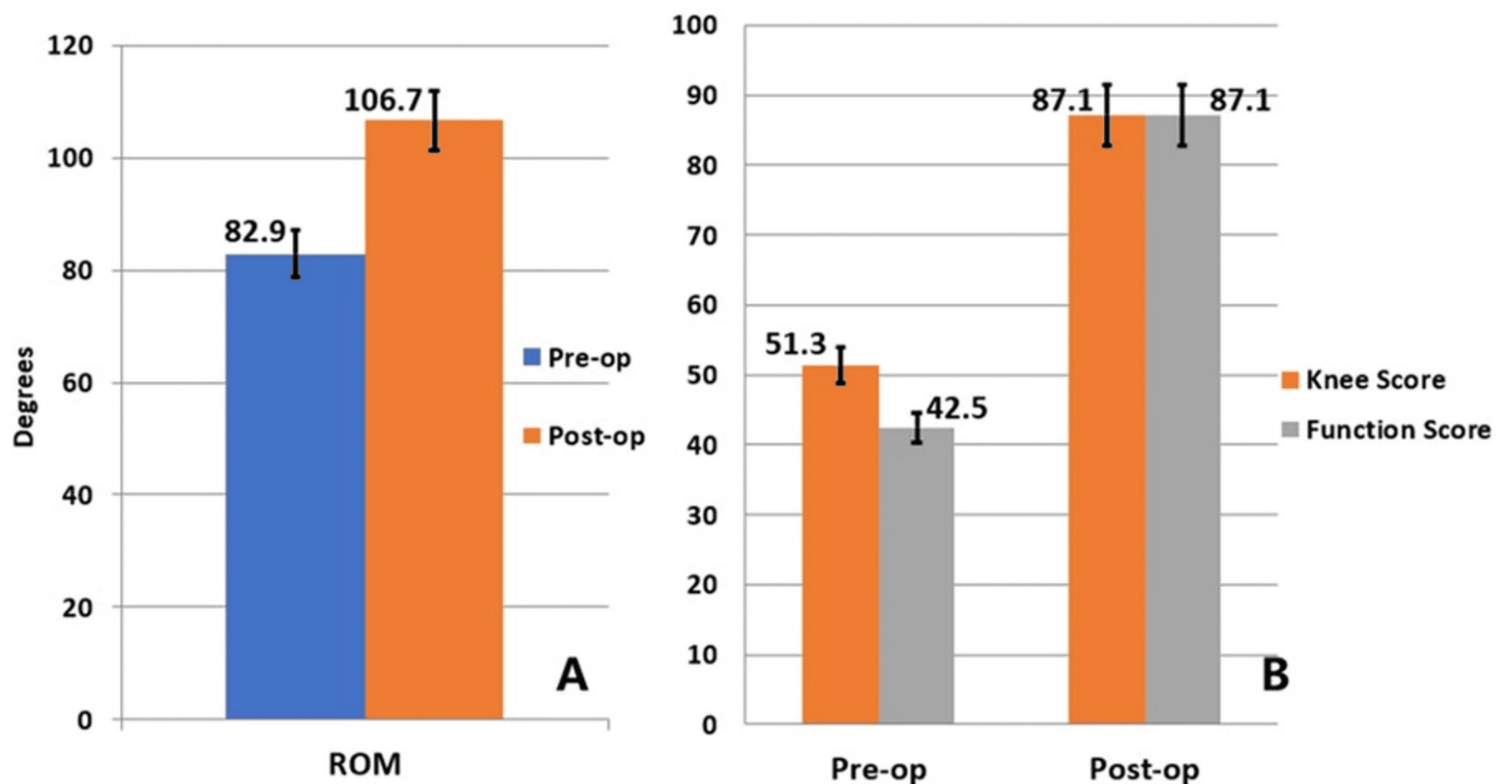
Changes in knee motion and function following conversion TKA A: the angle of motion of the knee increased significantly after surgery, from an average of 82.9 degrees (ranging from 70 to 100) before surgery improved to 106.7 degrees (ranging from 90 to 120) postoperatively; B: after the conversion TKA, either the knee score or the functional score substantially improved. TKA: total knee arthroplasty; ROM: range of motion

The mean postoperative HKA angles were 180.2^o^ (ranging from 174^o^ to 185^o^). Early recoveries and improvements in the range of motion were observed during follow-ups. All patients showed improvements in their knee motion and functional scores after surgery (Figure 3). An electronic chart review showed that no patients had unexpected emergency department visits within 30 days, readmissions, or site-specific complications throughout the outpatient follow-ups. Radiographic evaluation at the latest clinic visit showed no signs of radiological loosening and related implant revisions.

## Discussion

Performing conventional TKA on patients with severe post-traumatic knee arthritis is a great challenge. Prior retained periarticular implants, residual extra-articular deformity, and even deformed bony canals can all hinder the use of traditional intramedullary guiding systems to achieve accurate limb alignment [5]. In these complex situations, some extramedullary guiding methods, including intraoperative navigation or patient-specific instruments, have been reported to be valuable for performing TKA without removing the prior implants [5,9,14,15]. In this study, the authors propose a simplified yet feasible extramedullary guiding method as an alternative for primary TKA in arthritic knees with retained hardware when computer-assisted devices are not available.

Total knee arthroplasty (TKA) in the setting of previous periarticular hardware, known as conversion TKA, has been reported to increase resource utilization, readmissions, mechanical complications, infectious complications, and overall revision rates [2,4,16-18]. During conversion TKA, there is still ongoing debate regarding the simultaneous or staged removal of periarticular implants [19]. Kreitz et al. evaluated patients who required periarticular hardware removal prior to TKA, and their cohort comprised cases involving distal femoral and proximal tibial hardware from fractures and corrective osteotomy [16]. Their results exhibited an increased rate of readmissions and repeat procedures compared to matched primary TKA patients. By contrast, the retention of hardware does not seem to mitigate this risk profile. Manrique et al. studied patients with retained or partially retained periarticular hardware undergoing TKA, reporting increased postoperative and mechanical complications as well as stiffness [17]. Based on conventional minimally invasive concepts, the authors prefer EM methods for conversion TKA due to the increasing number of elderly patients and comorbidities less suitable for multiple surgeries or extended operation times. In this series, only the hardware interfering with bony preparation (mostly screws) was removed through a small incision at the site of a previous surgical wound, while most major implants were retained to save time and minimize invasiveness (Table 1, 2).

In the literature, recent advances in computer-assisted techniques, including computer-assisted navigation (CAN) and pre-made patient-specific blocks, made the extramedullary-referencing TKA more feasible for achieving accurate alignment and less invasive procedures [20]. However, they are not free of cost or complications. For example, the prolonged surgical duration due to the tracker setup and registration process, the steep learning curve, and the expenditure on the equipment impede their widespread adoption and acceptance [21,22]. Compared to the literature on CAN TKAs with retained implants [5,8,9,15,23,24], to our knowledge, our study is the first to specifically demonstrate the usefulness of freehand techniques for proximal tibia and distal femur cuts in these complex TKAs with retained hardware.

Based on prior experiences with navigation-assisted total knee arthroplasties, the authors recognized the importance of computer-assisted extramedullary positioning and the continuous, real-time verification necessary to maintain surgical accuracy [11,25]. Additionally, they confirmed that senior surgeons can achieve comparable precision in determining and performing bone cuts in conventional TKAs without the use of CAN technology [11]. Moreover, adhering to the fundamental requirement of ensuring enough parallel space between the distal femur and proximal tibia for prosthesis implantation, experienced surgeons generally find it straightforward to achieve this. They utilize dependable and stable oscillating saws, following careful preoperative planning and intraoperative verification principles. In this series, a corrected alignment with a deviation of less than 3 degrees was achieved in most cases (10 out of 12, 83%) after surgery, even though the initial deformity of the lower limb varied from 25 degrees varus to 10 degrees valgus. Similar accuracy was reported by Jenny et al. in their navigated TKAs in 2001, and our past experiences in navigation TKA, supporting the reliability of our proposed EM method in this study [25,26].

Similar to conventional CAN steps, accurate proximal tibial osteotomy is critical in the proposed method in this study. Preoperative imaging that helps plan the shape of the osteotomy, including long-leg films and lateral projections, is essential for later intraoperative verification. Additionally, the direction of the tibial ridge during knee flexion can be perpendicularly referenced to determine the accuracy of the tibial osteotomy. Finally, the complete contact between the distal femur and tibia following osteotomies can be used to verify whether limb alignment is skewed. Subsequently, similar concepts and verifications, along with parallel space relationships, were applied to the distal femoral osteotomy. Following these two technical adjustments, the surgeons can proceed with the routine steps in their daily practice of conventional TKAs, including component sizing, four-in-one femoral cuts (referencing the Whiteside’s Line), patellar resurfacing, and so on.

In conversion TKA, identifying and concurrently removing retained hardware can be time-consuming. In our series, a mildly extended but acceptable surgical duration of 101.2 minutes (ranging from 73 to 160 minutes) was required to complete the index procedures. Using the EM reference method, our patients were able to get simple prostheses implanted in primary TKAs with only a small amount of periarticular hardware being removed. This saved time that would have been spent finding and removing major hardware. In recent literature about conversion TKA, Steven Denyer et al. reported the mean operation time was 124.3 min, which was longer than that in our series [27].

**Figure 4:**
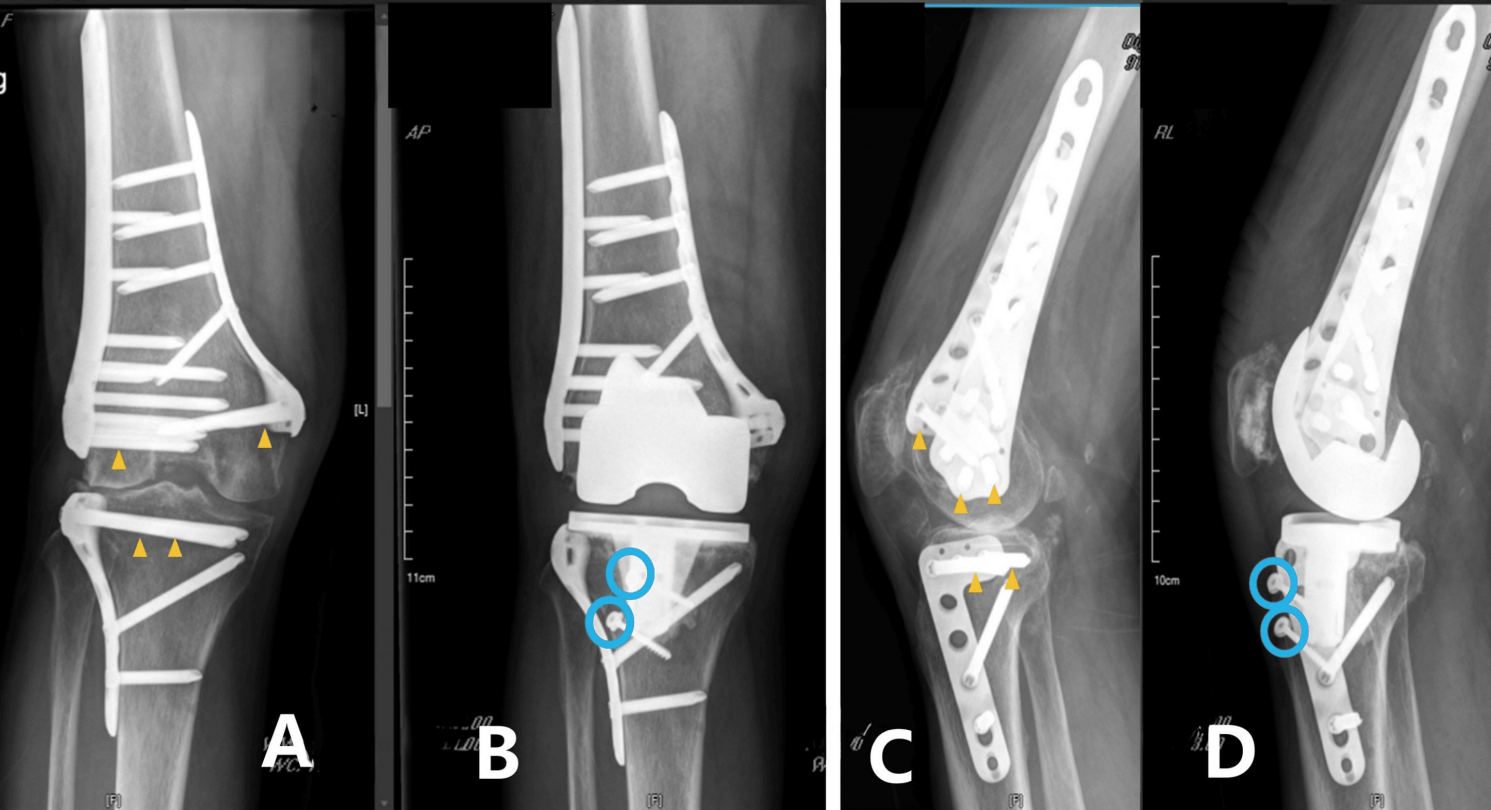
Conversion total knee arthroplasty with minimal periarticular hardware removal (1) A 73-year-old female suffered a floating knee trauma that was treated with locking plates on both sides. Post-traumatic knee arthritis developed two years later with a valgus deformity. During surgery, following CT identification, the blocking screws (yellow arrow) were removed, while two additional screws (hollow blue circles) were added to fix the tibial tubercle osteotomy (TTO) to facilitate exposure to the stiff knee. A, B: radiographic anterior-posterior (AP) view before and after surgery; C, D: lateral projections before and after surgery.

**Figure 5:**
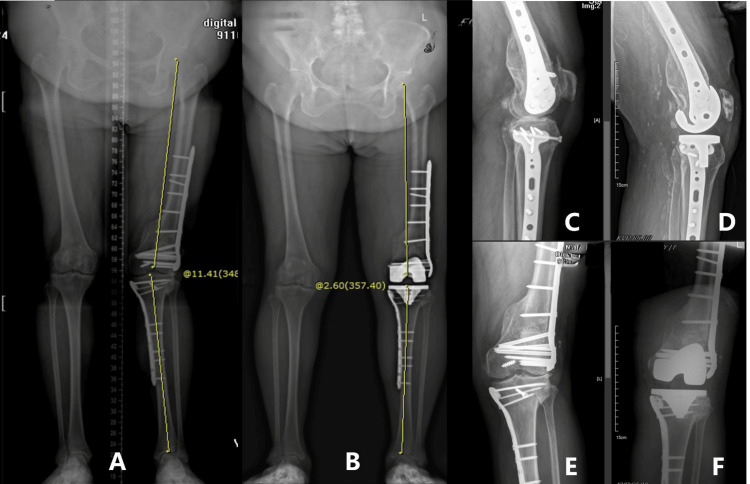
Conversion total knee arthroplasty with minimal periarticular hardware removal (2) A, B: scanograms before and after surgery illustrate the changes in lower limb alignment; C, D: lateral projections before and after surgery; E, F: radiographic AP view before and after surgery. A 69-year-old female with diabetes experienced post-traumatic knee arthritis after three knee injuries, leading to a valgus deformity (A). Mechanical alignment of the lower limb was restored (B), and relatively simple prostheses used in primary total knee arthroplasty were implanted with limited removal of retained hardware on both sides (B, D, & F).

Similar to most reported series, a variety of periarticular hardware was included in our cases. Although the heterogeneity of the cases may be questioned [28], it demonstrates the broad applicability of our method. Compared to retained nails, more fixation screws on plates crossing the metaphyseal area required timely identification and concurrent removal during surgery. In our most challenging cases, which involved locking plates on both sides of the knee (Cases 5 and 10, Figures 4, 5), a preoperative CT scan was arranged to obtain detailed spatial information about potential obstructive implants, helping to minimize hardware removal. In addition, additional surgical steps, such as tibial tubercle osteotomy, were taken into consideration for patients with preoperative stiffness or complex retained hardware (Case 10) [6]. 

This study has some inherent limitations, including its retrospective nature, relatively short follow-up period, limited sample size without a control group, and even the precision of the imaging evaluation tools. Due to the rarity of such complex knee conditions, we don't think it is fair to compare these cases with conventional intramedullary (IM)-guided or CAN-assisted TKA due to the complexity of the surgical exposure and the rarity of individual cases. Similar occurrences have been reported in Hernandez’s series (1.2%) [14]. Restoration of the neutral mechanical axis is believed to ensure the durability of the implanted prosthesis [29]. In the present study, the mechanical axis and component positions were evaluated using scanograms and standard lateral radiographs. Imaging errors might occur in cases of flexion deformity and rotation of either the femur or tibia [30]. Further evaluation with computed tomography helps address these issues and verify the actual alignment and the positions of the prostheses. However, concerns about radiation exposure and limitations for the insurance system prevent the routine use of computed tomography in our image follow-ups. In some series of conversion TKAs, a higher trend of mechanical failures, including periprosthetic fractures and loosening, has been reported [4,18]. However, such results were not observed in some series [19], including ours. We believe that these contrasting results validate the clinical diversity of conversion TKA, which necessitates preoperative case-by-case evaluation and individualized medical and postoperative rehabilitation plans.

We acknowledge that our approach may not be suitable for junior surgeons who have not yet mastered the concept of EM referencing or the verification of preoperative imaging planning with intraoperative anatomical structures. However, to our knowledge, there is currently limited literature reporting the outcomes of using a relatively simple EM method and fewer cutting jigs to perform total knee arthroplasty (TKA) with retained implants. By preserving the medullary canal and retaining implants, we aim to achieve clinical benefits such as reduced surgical trauma, blood preservation, and faster recovery without compromising operation time and necessary limb alignments. To verify the efficacy and reproducibility of the proposed method, further studies are warranted to compare its daily practice in primary TKAs to the conventional IM-guided or CAN-assisted ones.

## Conclusions

Our results suggest that the EM method proposed for conversion TKA effectively treats post-traumatic knee arthritis with remnant hardware, reducing surgical trauma and operation time. Short-term clinical benefits, including blood conservation, short hospital stays, and fast recovery, can be achieved while preserving optimal limb alignment.
